# Multiple Abscess of the Liver

**Published:** 1903-07

**Authors:** E. L. Ruffner

**Affiliations:** Assistant Surgeon, U. S. Army. Base Hospital, Iloilo, Panay, P. I.


### CLINICAL REPORT.

### Multiple Abscess of the Liver.

By E. L. RUFFNER, M. D.,
First Lieutenant and Assistant Surgeon, U. S. Army.

C. A. B., ex-soldier, inspector of customs for the Philippines, at Iloilo, Panay, P. I., aged 35; had lived in the tropics for about three and one-half years, or from the first occupancy of Iloilo by the American forces, as a soldier and in the customs department. He was first admitted to the military hospital at Iloilo, May 30, 1902, complaining of what was diagnosticated as acute pleurisy, right side, lower chest. He was in the hospital for ten days, being discharged June 10, 1902.

There was nothing to call attention to the bowel at the time. After being out about his business for about two months, as inspector of customs, he reëntered the hospital. This time he was suffering from pleurisy with effusion in the same area as that affected at the first admission—namely, the lower lobe of the right lung. He was in the hospital but one week, leaving this time at his own volition against advice.

December 12, 1902, he reëntered the hospital with pain and distress in the same place. Examination of the right chest showed dulness to flatness to third interspace in nipple line and corresponding position behind. No tenderness over the abdomen, urine normal, bowels having nothing to call attention to them, a cathartic being necessary. Liver found not enlarged, some cough, expectoration containing pus. After a few days in hospital, the temperature became such as to indicate pus. Leucocytosis present; malarial infection absent.

Exploratory aspirations were made in the chest at the ninth intercostal space, right side, mid-axillary line. Result unsatisfactory. This was repeated in a different area once again. It was thought at the time that it was because of a sacculated condition of the pleura that the results were so unsatisfactory. The patient's condition became steadily worse. The abdomen became tense, with tenderness over the hepatic area; the right side was chosen to lie upon as giving the most comfort at this time. Pain was complained of, radiating toward the right shoulder, of a dull, aching character. During this entire time the bowel had given no indication of trouble, and repeated questioning as to whether he had dysentery or bowel trouble during his long residence in the tropics, always gave the answer that he had never been troubled with them.

Preparations for operation for abscess of the liver were made. January 8, anesthetic given. But before exploratory puncture of the liver, I passed a large trocar into the pleural cavity at seventh interspace, mid-axillary line. Pus was found. I then did an exsection of two inches of the eighth rib. A large quantity of pus was evacuated. Drainage was left. Cultures of the pus did not show presence of ameba dysenteria. Side dressed twice a day, discharge getting less and less. On January 27, 1903, a diarrhea commenced. Examination showed numerous amebæ dysenteria present, and upon irrigation of chest with normal salt solution water would not return, nor was there any more discharge from drainage tube. An odor was also noticed in the opening in the chest wall.

February 3, 1903, patient died. Postmortem examination showed omentum small and adherent to the abdominal wall right iliac fossa. An old inflammatory tract was found, extending from cecum at the right iliac fossa up to the abdominal wall toward and to the liver. The colon and cecum between the ascending colon and abdominal wall were sacculated and surrounded by adhesions in the right iliac fossa, evidently an old inflammatory condition. Upon tearing this loose, a pus cavity was found communicating with the gut and filled with old amebic ulcers. The appendix was normal.

Liver not enlarged to any great extent, adherent to pleura, right side, surface presenting scars. Large abscess, which had been drained by the opening into the pleural cavity. Upon section, liver found to contain a number of small abscesses. Amebæ dysenteria not found in them.

Pleural cavity, heart, left lung and left pleura normal. Right lung very shrunken and pressed into small area about its root. The lung with adhesions divided the chest into two cavities, the only communication being around the apex. Both cavities were empty.

An explanation of the foregoing conditions might be offered as follows: starting from an original abscess of the liver, undoubtedly due to amebic infection, rupture into the right pleural cavity took place and, not being able on account of adhesions to go clear up the chest wall, the pus burrowed down the right flank between the ascending colon and the abdominal wall, being walled in by adhesive inflammation, until it reached the right iliac fossa, the intestine being anchored and the line of least resistance, it emptied into the gut. It would thus account for the onset of the amebic dysentery five or six days before death, and the fact that the irrigation did not return from the opening in the chest and the character of the odor from the chest wound. which at this time was in communication with the intestinal tract, would further justify this conclusion.

That the patient, with this serious amount of damage, should suffer little pain and be able to work and keep upon his feet until five weeks before his death, is another strange feature of the case. A practical deduction from this case is that one should always suspect and look for abscess of the liver in tropical countries in any case presenting a pleurisy low down on the right side, even if that is the only symptom present.

BASE HOSPITAL, ILOILO, PANAY, P. I.

---

MANAGEMENT OF PREMATURE LABOR.—The usual precautions taken in normal cases should be observed, says E. P. Davis, with special care to avoid rupture of the membranes. When dilatation is but partly complete, use elastic bag or intrauterine tampon of aseptic gauze. If placenta is retained, antiseptic delay may be practised until spontaneous expulsion, if no hemorrhage occurs. If bleeding is present, anesthetise, dilate uterus and remove placenta completely.—*Medical Herald.*

